# Classifying Chronic Kidney Disease Stages Based on Predicted Value of Estimated Glomerular Filtration Rate From Serum Creatinine Levels Using Chronic Kidney Disease Risk Factors and Renal Sinus Anteroposterior Diameter on a Computed Tomography Image

**DOI:** 10.7759/cureus.73085

**Published:** 2024-11-05

**Authors:** Yasuhiro Inokuchi, Tsuneyuki Takashina, Yusuke Hayashi, Jo Sakihara, Masahiro Uematsu, Hiromasa Kurosaki

**Affiliations:** 1 Department of Radiology, Edogawa Hospital, Tokyo, JPN; 2 Department of Radiology and Radiation Oncology, Edogawa Hospital, Tokyo, JPN

**Keywords:** chronic kidney disease (ckd), cutoff value, estimated glomerular filtration rate (egfr), multidetector row computed tomography (mdct), renal sinus fat invasion

## Abstract

Objective: This study aimed to investigate whether the calculated predicted value of the estimated glomerular filtration rate from serum creatinine levels (eGFRcreat-PV) from multiple regression equations using the anteroposterior diameter of the renal sinus (APDRS) measured on multidetector row computed tomography (MDCT) axial images and easily obtainable chronic kidney disease (CKD) risk factors (age and body mass index, BMI) is useful for separating CKD stages.

Material and methods: The Review Board approved this study, and the additional approval requirement was waived for this retrospective study. We investigated the association between eGFRcreat and age, BMI, and APDRS using stepwise multivariate regression analysis in 98 patients who underwent MDCT and calculated eGFRcreat-PV from the obtained multiple regression equation. We investigated significant differences in eGFRcreat-PV according to the variation in the CKD stage 3 categories and cutoff value. Additionally, we analyzed the area under the curve (AUC), sensitivity, and specificity of eGFRcreat-PV to differentiate between the patients with greater and less than CKD stage 3.

Results: Age, BMI, and APDRS scores were independent factors influencing eGFRcreat. The difference in eGFRcreat-PV between the groups with greater and less than CKD stage 3 was significant. Additionally, the cutoff value, AUC, sensitivity, and specificity of eGFRcreat-PV used to distinguish between the two groups of greater and less than CKD stage 3 were 61.5, 0.801, 63.9%, and 80.6%, respectively.

Conclusion: The eGFRcreat-PV from the APDRS, measured on MDCT axial images, along with age and BMI, helps differentiate the less and more than CKD stage 3.

## Introduction

Chronic kidney disease (CKD) is a prevalent condition worldwide [1,2]. It is associated with metabolic syndrome [3,4] and has various complications, of which the risks of end-stage kidney disease (ESKD) and cardiovascular disease are the highest [5,6]. ESKD is caused by severe renal function reduction. Complete recovery of renal function is not expected in patients with ESKD requiring dialysis or kidney transplantation for survival [7,8]. Therefore, early discovery and treatment of CKD are necessary for risk management of various CKD complications [1,9]. CKD is divided into five stages [10]. The risk of complications and progression to ESRD is significantly increased with disease progression to CKD stage 3 (estimated glomerular filtration rate from serum creatinine levels, eGFRcreat-PV, <60 mL/minute/1.73 m^2^). Therefore, monitoring renal function to reduce complication risk is crucial. CKD is currently diagnosed based on patients' eGFRcreat and urinary albumin excretion rates [11-13]. The role of imaging in CKD diagnosis is to observe abnormal kidney morphology [14]. Although ultrasonography and nuclear medicine scans can be utilized for renal function diagnosis [15,16], these imaging techniques have reduced accuracy in assessing renal function [17]. Thus, image analysis is not the first choice for renal function evaluation [11-13]. However, renal parenchymal analog-to-digital converter (ADC) values on MRI have recently been reported to be useful in monitoring renal dysfunction [18-20]. Foster et al. [21] reported that the measurement of renal sinus fat (RSF) volume using multidetector row computed tomography (MDCT) imaging is a useful method for evaluating renal function, and RSF has been recognized as an independent factor affecting renal function. Furthermore, several previous studies [22-24] have indicated that RSF accumulation leads to structural and functional changes in the kidney and renal vessels. We previously reported that the anteroposterior diameter of the renal sinus (APDRS) measured on axial MDCT images is a simple alternative method for estimating RSF accumulation [25]. In addition, APDRS reflects RSF and arteriosclerosis-induced renal atrophy; therefore, APDRS was found to be more strongly correlated with eGFRcreat than with RSF [25]. Thus, this study aimed to investigate whether eGFRcreat can be estimated and CKD staging can be performed using easily obtainable risk factors for CKD (age and body mass index, BMI) [26] and APDRS.

## Materials and methods

Patient characteristics

**Figure 1:**
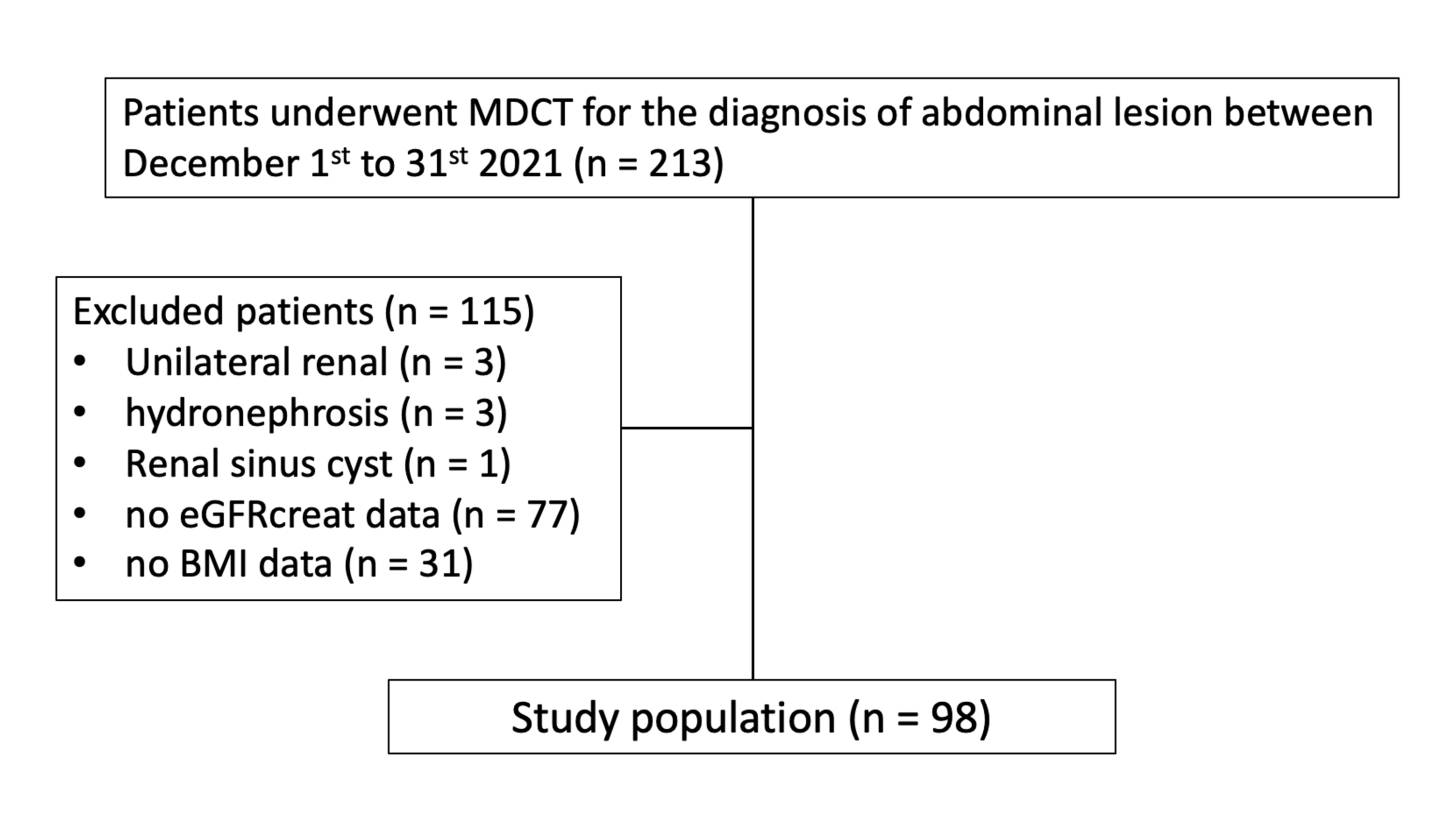
Flowchart of patient enrollment MDCT: multidetector row computed tomography; eGFRcreat: estimated glomerular filtration rate from serum creatinine levels; BMI: body mass index

This study retrospectively selected 213 consecutive patients who underwent abdominal imaging using 64-slice MDCT between December 1 and 31, 2021 (Figure 1). The Review Board approved this study, and the requirement for informed consent was waived for this retrospective study. We excluded seven patients with unilateral renal disease (n = 3), hydronephrosis (n = 3), and renal sinus cyst (n = 1) because of the possible effect of these conditions on the measurement of APDRS. In addition, we excluded 108 patients without eGFRcreat (n = 77) and BMI data (n = 31) because of the possible effect of these conditions on the quantitative analysis of this study. After exclusion, this study included 98 patients (63 men and 35 women; age range: 24-89 years; mean: 67 years) with BMI, APDRS, serum creatinine, and eGFRcreat ranging from 13.7 to 32.9 kg/m^2^ (mean: 24.4 kg/m^2^), 7.2 to 44.1 mm (18.2 mm), 0.36 to 13.79 mg/dL (1.03 mg/dL), and 3 to 133 mL/minute/1.73 m^2^ (66.8 mL/minute/1.73 m^2^), respectively.

CT protocol

CT scanner (Revolution EVO, GE Healthcare, Chicago, IL) equipped with a 64-slice detector was used. CT parameters were as follows: tube voltage, 120 kVp; tube current, automatic tube current modulation program; collimation, 0.625 mm; detector configuration, 64 × 0.625 mm; noise index, 13; pitch factor, 0.984:1; and gantry rotation time, 0.5 seconds. All transverse CT images were reconstructed at 5 mm-thick sections, with the intensity of the adaptive statistical iterative reconstruction set at 50%. Computed tomography (CT) was performed in the cephalocaudal direction, and all images were obtained from the top of the liver to the bottom of the ischium.

Measurement of APDRS

**Figure 2:**
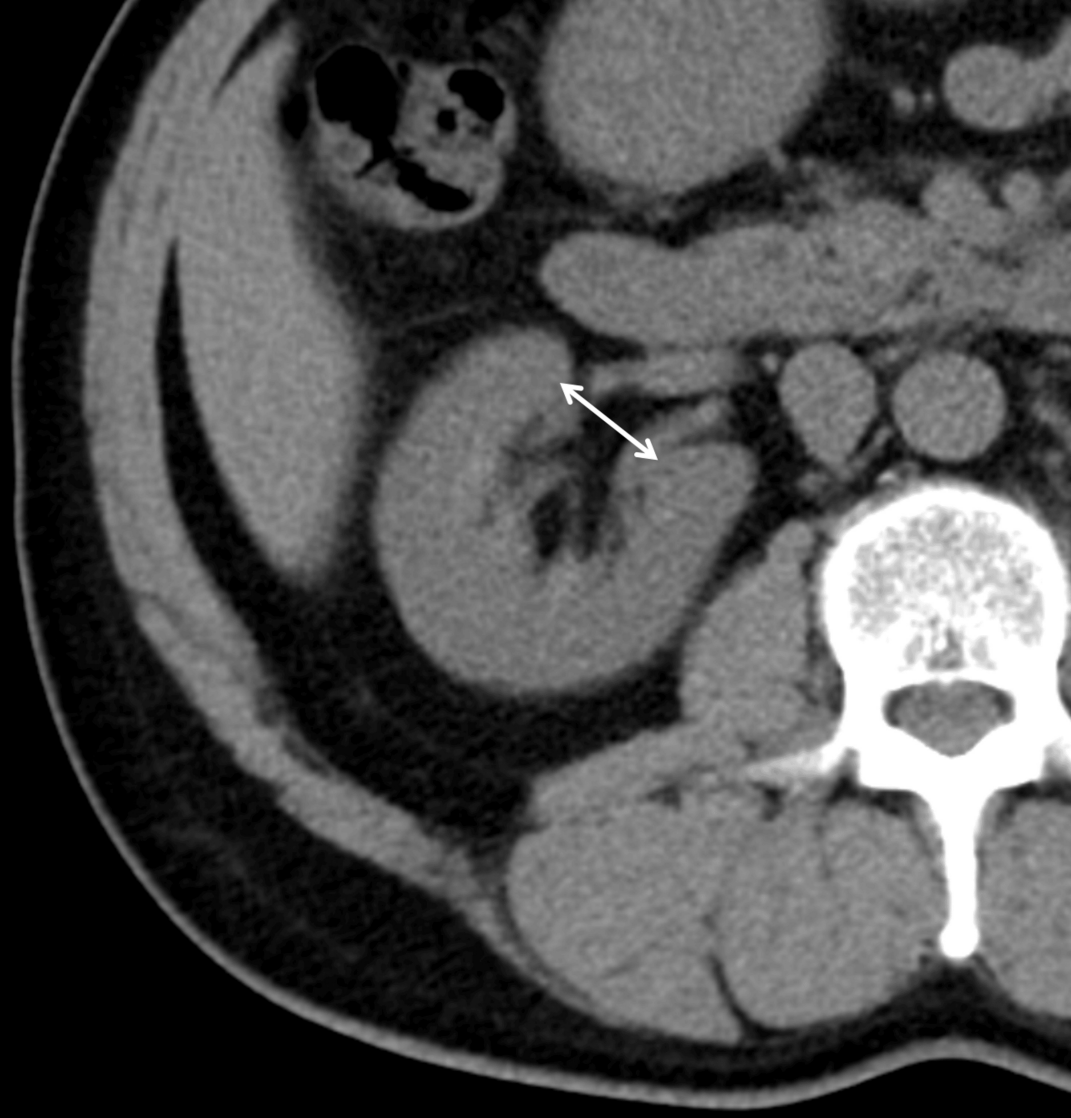
Measurement of APDRS APDRS: anteroposterior diameter of the renal sinus

The slice for measurement was selected from the center of the entire renal hilum and rendered on an axial CT image. The APDRS was defined as a line connecting the ventral and dorsal side edges of the inner cut edge between the right renal sinus and the renal hilum in the axial CT image (Figure 2). If the total range was an even number of slices, a larger APDRS score was selected for the two center slices. A radiological technician with 20 years of experience in MDCT imaging measured the APDRS scores of all patients using a measurement tool on a picture archiving and communication system monitor (Synapse; FUJIFILM, Tokyo, Japan).

Statistical analysis

Stepwise multivariate regression analysis with eGFRcreat as an objective variable was used to investigate the association between eGFRcreat and easily obtainable risk factors for CKD (age and BMI) and APDRS. In addition, we calculated the predicted values of eGFRcreat (eGFRcreat-PV) using the obtained multiple regression equation. The 98 patients in this study were classified at each stage according to the evidence-based clinical practice guidelines for CKD 2023 of the Japanese Society of Nephrology [10]. We investigated the difference in the eGFRcreat-PV between less and more than stage 3 categories using a two-sample t-test and evaluated the optimal cutoff value and diagnostic accuracy (sensitivity, specificity, and the area under the receiver operating characteristic, ROC, curve) from ROC analysis using the Youden index to investigate whether predicted values can be used as a simple new indicator for separating the patients of less and more than stage 3 categories. The threshold for statistical significance was set at p < 0.05. All statistical analyses were performed using the Easy R software (Saitama Medical Center, Jichi Medical University, Saitama, Japan).

## Results

Age, BMI, and APDRS scores were independent factors that influenced eGFRcreat (Table 1).

**Table 1:** Factors associated with eGFRcreat in stepwise multivariate regression analysis Adjusted R^2^ = 0.2901 eGFRcreat (male) = 194 × creatinine - 1.094 × age - 0.287 eGFRcreat (female) = 194 × creatinine - 1.094 × age - 0.287 × 0.739 BMI = weight (kg) / height (m) × height (m) STD: standard deviation; BMI: body mass index; APDRS: anteroposterior diameter of the renal sinus; eGFRcreat: estimated glomerular filtration rate from serum creatinine levels

Subjects	Estimate STD	Error	t value	p values
Intercept	153.2780	16.5429	9.265	<0.001
Age (year)	-0.5452	0.1485	-3.671	<0.001
BMI (kg/m^2^)	-1.5205	0.5739	-2.649	0.009
APDRS (mm)	-0.6959	0.3317	-2.098	0.038

The multiple regression equation obtained for eGFRcreat-PV is as follows: -0.5452 × age - 1.5205 × BMI - 0.6959 × APDRS + 153.2780. Table 2 shows patient characteristics corresponding to less and more than CKD stage 3, based on the evidence-based clinical practice guidelines for CKD 2023 of the Japanese Society of Nephrology [10].

**Table 2:** Patient characteristics at each group Data are represented as mean ± standard deviation or n (%) p value was obtained with the two-sample t-test Age, BMI, creatinine, and eGFRcreat were derived from data obtained within one month before MDCT imaging APDRS: anteroposterior diameter of the renal sinus; BMI: body mass index; eGFRcreat: estimated glomerular filtration rate from serum creatinine levels; eGFRcreat-PV: predicted value of estimated glomerular filtration rate from serum creatinine levels; MDCT: multidetector row computed tomography

Subjects	eGFRcreat ≧ 60	eGFRcreat < 60	p values
Number of patients	62	36	-
Male	41 (67.1%)	22 (61.1%)	-
Female	21 (32.9%)	14 (38.9%)	-
Age (year)	62.8 ± 15.6	74.2 ± 9.6	<0.001
BMI (kg/m^2^)	23.6 ± 3.4	25.7 ± 3.8	0.007
APDRS (mm)	16.4 ± 6.0	21.4 ± 7.4	<0.001
Creatinine (mg/dL)	0.71 ± 0.14	1.57 ± 2.14	0.002
eGFRcreat (mL/minute/1.73 m^2^)	80.2 ± 15.3	43.7 ± 14.1	<0.001
eGFRcreat-PV	71.5 ± 12.1	58.6 ± 9.7	<0.001

**Figure 3:**
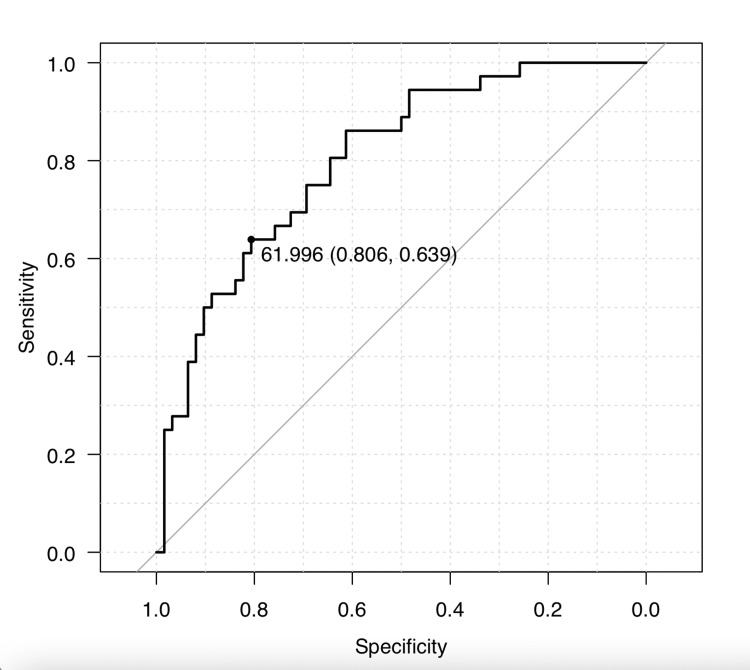
Graph showing an ROC curve for separating less and more than CKD stage 3 using eGFRcreat-PV ROC: receiver operating characteristic; CKD: chronic kidney disease; eGFRcreat-PV: predicted value of estimated glomerular filtration rate from serum creatinine levels

The difference in eGFRcreat-PV between the groups with greater and less than CKD stage 3 was significant (p < 0.001). The optimal cutoff value of eGFRcreat-PV calculated by ROC analysis for distinguishing between the less and more than CKD stage 3 categories was 61.9. Additionally, the sensitivity was 63.9%, specificity was 80.6%, and the area under the ROC curve was 0.801 (95% confidence interval, 0.713-0.889) (Figure 3) for determining diagnostic accuracy using this optimal cutoff value.

## Discussion

We obtained eGFRcreat-PV from multiple regression equations using age, BMI, and APDRS and found a significant difference between patients with CKD stages less than or more than 3 at eGFRcreat-PV. In addition, we demonstrated that the cutoff values and area under the curve for differentiating the CKD stage 3 categories were 61.9 and 0.801, respectively. eGFRcreat-PV was useful for separating fewer and more CKD stage 3 categories. Therefore, eGFRcreat-PV use makes it possible to simultaneously evaluate kidney function and morphology and offer beneficial information on CKD progression with just one CT examination. This includes the similarity between the factors used to calculate eGFRcreat and eGFRcreat-PV. Age was used in both formulas. Further, obesity increases renal blood flow and glomerular hyperfiltration, leading to decreased renal function [27,28]. Therefore, BMI may be a useful measure of eGFRcreat. Although two different indicators of creatinine and APDRS were used for the respective calculation of eGFRcreat and eGFRcreat-PV, those were caused in part by a common phenomenon. Creatinine is a waste product filtered by the glomeruli of the kidney and excreted in the urine. However, renal diseases interfere with creatinine filtration into the glomeruli, causing elevated creatinine levels in the blood. In addition, renal diseases have been shown to cause kidney atrophy as they progress to end-stage renal failure [29]. As APDRS enlargement is due to increased RSF and kidney atrophy [25], kidney atrophy is recognized as a factor directly affecting APDRS readings. Age, BMI, and APDRS were included as parameters of kidney aging, reflux, and fibrosis. Therefore, eGFRcreat P-V composed of these indicators may have been useful in identifying patients with CKD stage 3. We have not seen reports of such a method used to separate CKD stages using CT imaging information. If eGFRcreat-PV can be used to identify patients with CKD stage 3 or higher who are at an increased risk for various complications, we have the opportunity for early intervention. Appropriate treatment and lifestyle modifications at this stage can delay further decline in renal function. Several recent studies have used MRI-based renal ADC values. However, a standardized protocol has not yet been established for this value. It is concerning that variations in ADC values among different manufacturers and imaging sequences may affect the cutoff value [18,20,30]. The eGFRcreat-PV used in this study is a combination of age and BMI, which are easily obtainable indices and values traced on CT images. Therefore, the effect of variation among different CT models can be eliminated. Furthermore, CT is cheaper and easier to perform than MRI. Therefore, this method using CT can provide adequate information for many patients, in contrast to MRI, which relies heavily on compliance owing to poor systemic status. This study had several limitations. First, this was a retrospective study with a small sample size. Second, the eGFRcreat-PV was not applicable in all patients. Patients with unilateral kidneys, cysts, or hydronephrosis that affected the APDRS measurement method were excluded. Therefore, further studies are required to investigate the indications for patients with such a renal disease.

## Conclusions

The eGFRcreat-PV calculated from the multiple regression equation of age, BMI, and APDRS measured on MDCT axial images was an easily obtainable indicator of eGFRcreat that may be able to distinguish the CKD stage 3 categories in patients.
